# Isthmin 1 is Expressed by Progenitor-Like Cells in the Lung: Phenotypical Analysis of Isthmin 1^+^ Hematopoietic Stem-Like Cells in Homeostasis and during Infection

**DOI:** 10.1155/2022/2909487

**Published:** 2022-04-01

**Authors:** Guadalupe Rivera-Torruco, Carolina A. Martínez-Mendiola, Tania Angeles-Floriano, Gustavo Alberto Jaimes-Ortega, José Luis Maravillas-Montero, Rodolfo García-Contreras, Yolanda González, Esmeralda Juárez, Porfirio Nava, Vianney Ortiz-Navarrete, Oscar Medina-Contreras, Paula Licona-Limón, Ricardo Valle-Rios

**Affiliations:** ^1^Unidad Universitaria de Investigación, División de Investigación, Facultad de Medicina, Universidad Nacional Autónoma de México-Hospital Infantil de México Federico Gómez (UNAM-HIMFG), Mexico City, Mexico; ^2^Departamento de Fisiología, Biofísica y Neurociencias, Centro de Investigación y de Estudios Avanzados (CINVESTAV), Mexico City, Mexico; ^3^Laboratorio de Investigación en Inmunología y Proteómica, Hospital Infantil de México Federico Gómez (HIMFG), Mexico City, Mexico; ^4^Facultad de Química, Universidad Nacional Autónoma de México (UNAM), Mexico City, Mexico; ^5^Subdirección de Diagnóstico clínico y Departamento de Laboratorio Clínico, Hospital Infantil de México Federico Gómez, Mexico City, Mexico; ^6^Posgrado en Biología Experimental, Departamento de Ciencias Biológicas y de la Salud, Universidad Autónoma Metropolitana (UAM), Mexico City, Mexico; ^7^Red de Apoyo a la Investigación, Universidad Nacional Autónoma de México e Instituto Nacional de Ciencias Médicas y Nutrición Salvador Zubirán (INCMNSZ), Mexico City, Mexico; ^8^Departamento de Microbiología y Parasitología, Facultad de Medicina, Universidad Nacional Autónoma de México (UNAM), Mexico City, Mexico; ^9^Departamento de Investigación en Microbiología, Instituto Nacional de Enfermedades Respiratorias Ismael Cosío Villegas (INER), Mexico City, Mexico; ^10^Departamento de Biomedicina Molecular, Centro de Investigación y de Estudios Avanzados (CINVESTAV), Mexico City, Mexico; ^11^Unidad de Investigación Epidemiológica en Endocrinología y Nutrición, Hospital Infantil de México Federico Gómez (HIMFG), Mexico City, Mexico; ^12^Departamento de Biología Celular y del Desarrollo, Instituto de Fisiología Celular, Universidad Nacional Autónoma de México (UNAM), Mexico City, Mexico

## Abstract

The process by which blood cells are generated has been widely studied in homeostasis and during pathogen-triggered inflammatory response. Recently, murine lungs have been shown to be a significant source of hematopoietic progenitors in a process known as extramedullary hematopoiesis. Using multiparametric flow cytometry, we have identified mesenchymal, endothelial, and hematopoietic progenitor cells that express the secreted small protein Isthmin 1 (ISM1). Further characterization of hematopoietic progenitor cells indicated that ISM1^+^ Lineage^−^ Sca-1^+^ c-kit^+^ (ISM1^+^ LSK) cells are enriched in short-term hematopoietic stem cells (ST-HSCs). Moreover, most Sca-1^+^ ISM1^+^ cells express the residence marker CD49a, and this correlated with their localization in the extravascular region of the lung, indicating that ISM1^+^ cells are lung-resident cells. We also observed that ISM1^+^ cells express TLR4, TLR5, and TLR9, and, in a mouse model of sepsis induced by *P. aeruginosa*, we observed that all the LSK and ISM1^+^LSK cells were affected. We conclude that ISM1 is a novel biomarker associated with progenitor-like cells. ISM1^+^ cells are involved in the response to a bacterial challenge, suggesting an association between ISM1-producing cells and dangerous inflammatory responses like sepsis.

## 1. Introduction

Hematopoietic stem and progenitor cells (HSPCs) are a heterogeneous group of cell subpopulations that give rise to blood cells during the lifetime of an organism. In both humans and mice, the fetal liver is the main source of hematopoietic progenitors during gestation, before being replaced by bone marrow in adulthood. Hematopoietic stem cells (HSCs) derived from the bone marrow are enriched in the cellular fraction lacking cell-surface markers present in lineage-committed hematopoietic cells, but they express the stem-cell antigen 1 (Sca-1) and stem cell factor receptor (c-kit); these Lin^−^c-kit^+^Sca-1^+^ cells are identified as the LSK subset [1, 2]. This LKS fraction also contains the metabolically active subset of the HSCs known as multipotent progenitors (MPPs). Globally, HSPCs may be classified as long-term HSC (LT-HSC; LSKCD150^+^CD48^−^), short-term HSC (ST-HSC; LSKCD150^−^CD48^−^), MPP2 (LSK CD150^+^CD48^+^), or MMP3/4 (LSKCD150^−^CD48^+^) [3].

Hematopoiesis can also take place in the spleen [4], liver [5, 6], and lungs [7–9] under severe stress or pathological conditions in a process called extramedullary hematopoiesis (EMH) [10]. Most of these findings have been derived from radiological studies; there are several reviews that sum up these rare hematological phenomena [11]. However, recent evidence indicates that the lung [12, 13] and the small intestine [14, 15] support EMH under homeostatic conditions.

Current knowledge on EMH indicates that murine lungs are active hematopoietic organs, which can account for 50% of platelet production. Resident lung HSPCs exhibit the same phenotype as their counterparts in bone marrow and, importantly, they can produce both lymphoid and myeloid lineages [12, 13]. However, there is no additional information about molecular markers or mediators characterizing the cells responsible for lung EMH during homeostasis or inflammation.

ISM1 is a secreted protein characterized by the presence of two functional domains: a thrombospondin type 1 repeat (TSR1) and the adhesion-associated domain in MUC4 and other proteins (AMOP) [16]. *ISM1* is involved in embryonic hematopoiesis in zebrafish and is highly expressed in the respiratory track during development, both in chicken and mice [17, 18]. Nevertheless, it is still not known whether ISM1 is related to lung EMH.

We previously reported the initial identification of *ISM1*-expressing tissues in humans. *ISM1* is expressed in several human tissues such as the lung, skin, small intestine, and activated peripheral blood mononuclear cells (PBMC). In the mouse, *ISM1* is expressed at high levels in the respiratory track (trachea and lung), and initial studies also detected intracellular protein expression of ISM1 in subsets of T cells and NK cells [19]. However, detailed analysis about the cells expressing ISM1 in the lung is still missing.

In the present study, we describe that in murine lungs, ISM1 is present in progenitor cells resembling mesenchymal progenitor cells (MSC), endothelial progenitor cells (EPCs) and HSPCs. We also evaluated the tissue resident properties of ISM1^+^ cells. Moreover, we characterized changes in ISM1^+^ cells in the LSK compartment both in homeostasis and during infection with *P. aeruginosa.* These observations strongly suggest a role for ISM1 in the field of progenitor cells.

## 2. Materials and Methods

### 2.1. Animals and Ethics Statement

Male C57BL/6 mice (5-8 weeks old) were obtained from Envigo RMS S.A. All animal experiments were performed according to protocols approved by Mexican NOM-062-ZOO-1999 (SAGARPA) and in agreement with the Guide for the Care and Use of Laboratory Animals of the National Institutes of Health (NIH) and internal guidelines. The protocol was approved by the Mexican Children's Hospital Federico Gomez ethics committee.

This study was carried out with the approval and under the guidelines of the Institutional Review Boards (IRB) of the Ethics Committee of the Mexican Children's Hospital Federico Gómez.

### 2.2. Lung, Lamina Propria, Spleen, and Blood Single-Cell Preparation for Flow Cytometry

#### 2.2.1. Lung Digestion

Lungs from C57BL/6 mice were removed after intracardiac perfusion with 5 ml of cold phosphate-buffered saline 1x (PBS 1x), minced, and placed in 20 ml of RPMI medium containing 20 mg of Collagenase I and 10 mg of DNase I (Sigma). Samples were placed in an orbital shaker incubator (200 rpm) for 15 min at 37°C, mechanically disaggregated, strained through a 70 *μ*m mesh, then through a 40 *μ*m mesh, and centrifuged. The cell pellet was treated with erythrocyte lysis buffer, washed, and finally, resuspended in PBS 1x supplemented with 5% fetal bovine serum (FBS) prior to counting and stain.

#### 2.2.2. Lamina Propria Digestion

Small intestine was removed from C57BL/6 mice, intestinal content was flushed out and then minced in pieces of 2 cm long. These pieces were placed inside a 50 ml conical tube with 20 ml Hanks's buffer with 0.5 mM EDTA. This tube was placed in the orbital shaker incubator (250 rpm) during 20 min. at 37°C. Following this, the content of the tube was filtered with a stainless steel 20 mesh strainer; the cell tissue remaining in the strainer was washed with PBS 1x. The tissue was collected on a 50 ml conical tube with 20 ml of RPMI. The tissue was thoroughly minced with a pair of dissecting scissors. Then, 20 mg of Collagenase I and 10 mg of DNase I (Sigma) placed in an orbital shaker incubator (200 rpm) at 37°C for 20 minutes. The cell suspension was strained trough a 70 *μ*m mesh inside a new 50 ml tube, then centrifuged (5 min., 1500 rpm). The supernatant was discarded, and the pellet was treated with 5 ml of erythrocyte lysis buffer during 5 min. at 4°C. Following this, 5 ml of cold PBS1x were added to the pellet and then centrifuged (5 min., 1500 rpm). The pellet was resuspended in approximately 500 *μ*l in PBS 1x with 0.5 mM EDTA and supplemented with 5% FBS prior to count.

#### 2.2.3. Blood Recovery

Blood was recovered by simultaneous perfusion of 1x PBS with 0.5 mM EDTA and retrieved with an insulin syringe through the abdominal aorta. Blood was recovered inside a 15 ml conical tube to a volume of 10 ml (with PBS 1x 0.5 mM EDTA), then centrifuged (5 min., 1500 rpm). The supernatant was discarded, and the pellet was incubated with 5 ml of erythrocyte lysis buffer during 5 minutes at 4°C. Following this, 5 ml of PBS 1x were used to wash the cells and then centrifuged (5 min., 1500 rpm). Cellular pellet was finally resuspended in approximately 500 *μ*l of PBS 1x supplemented with 5% FBS prior to count.

#### 2.2.4. Bone Marrow Recovery

Bone marrow cells were flushed from the femurs and tibias into 1x PBS with 0.5 mM EDTA using a syringe and a 21-Gauge needle. Residual bone marrow red blood cells were lysed with erythrocyte lysis buffer, washed, and resuspended in PBS 1x supplemented with 5% FBS prior to count.

### 2.3. Flow Cytometry

Flow cytometry was performed using the following monoclonal antibodies: AF700 anti-CD45, PercP anti-CD45, PercPCy5.5 anti-CD19, PercPCy5.5 anti-TER119, PercPCy5.5 anti-CD8a, PercPCy5.5 anti-NK1.1, PercPCy5.5 anti-FceR1a, PercPCy5.5 anti-CD34, PercPCy5.5 anti-EpCAM, APC anti-Sca-1, FITC anti-Sca-1, PECy7 anti-c-kit, APC anti-ST2, APC-Cy7 anti-NK1.1, FITC anti-CD31/PECAM, PacificBlue anti-flk1/VEGFR2/KDR, VB605 anti CD140a/PDGFR, FITC anti-CD49d, APC anti-CD49a, PECy7 anti-CD49b, APC-fire 750 anti-CD48, VB605 anti-CD150, FITC anti TLR9 (CD289), AF647 anti TLR5 (CD285), PECy7 anti TLR4 (CD284), PE anti-ISM1, and PE anti-IgG2b all from Biolegend (San Diego, CA) and PercePCy5.5 anti-CD3e, PercPCy5.5 anti-Ly6G, PercPCy5.5 anti-CD11b, and VF450 anti-CD127 from TONBO (San Diego, CA). Live cells were detected using Zombie Violet Fixable Viability Kit from Biolegend or ViaKrome 808 Fixable Viability Dye from Beckman Coulter (Table S1). Data acquisition was performed on CytoFLEX LX Flow Cytometer (Beckman Coulter) and analyzed using FlowJo v10.7.2.

### 2.4. ImageStream

We harvested 2 × 10^6^ freshly isolated lung cells as described above, and the cells were stained with the extracellular markers FITC anti-Sca-1 and APC anti-CD45 followed by intracellular staining with PE anti-ISM1 and DRAQ5 for nuclear staining (All from Biolegend). Images were captured using the Amnis ImageStream Mark II Imaging Flow Cytometer with 60x magnification (EMD Millipore). Data were acquired using Amnis INSPIRE software and analyzed using Amnis *IDEAS* software.

### 2.5. Extravascular Localization of ISM1^+^ Cells

To test for extravascular localization (tissue residency), mice were injected intravenously with APC anti-Sca-1 antibody 5 min before lung cell recovery as indicated before. Isolated cells were stained with FITC anti-Sca-1 and intracellular PE anti-ISM1 antibodies followed by flow cytometry acquisition.

### 2.6. Pseudomonas Aeruginosa Infection

C57BL/6 were infected by intraperitoneal injection of 2 × 10^7^ CFU of *Pseudomonas aeruginosa* strain UCBPP-PA14 diluted in 200 *μ*l of sterile PBS 1x. 8 h postinfection, mice were euthanized, and blood, bone marrow, and lungs were recovered. Cells were analyzed by flow cytometry. Plasma was recovered and stored at -80°C.

### 2.7. Toll-Like Receptor Stimulation in Lung Cells

Following lung digestion, cells were recovered in RPMI 1640 (Caisson) with FBS 10%, antibiotic 1x (Sigma), and L-glutamine solution (Sigma) and were seeded in a 48-well plate (1 × 10^6^ cells per well). TLR ligands LPS-EK, FLA-ST, and ODN1826 were purchased from InvivoGen. LPS-EK final concentration in media was 500 ng/ml, FLA-ST 10 ng/ml, and ODN1826 5 *μ*M. The plates were incubated during 4 h at 37°C and 5% CO_2_. Following incubation, cells were recovered for flow cytometry protocol.

### 2.8. Statistics

Mouse data are reported as mean ± SEM and were analyzed by unpaired Student's *t*-test, whereas multigroup comparisons were performed using a one-way ANOVA test and Bonferroni's post hoc. GraphPad PRISM version 7.0, GraphPad Software Inc. (La Jolla, CA), was used for all these analyses.

## 3. Results

### 3.1. SM1^+^ Cells Are Enriched in the Lung and Express Markers of Stem Cells

**Figure 1:**
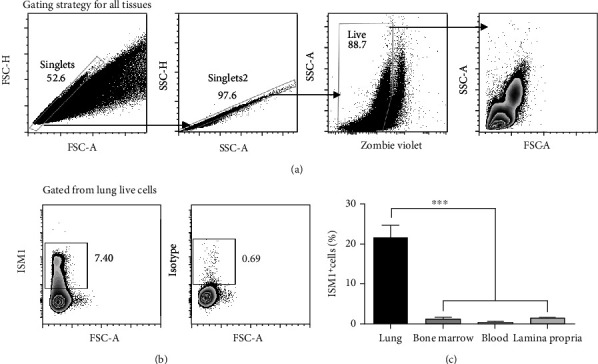
ISM1^+^ cells are enriched in lung. (a) Living cells were isolated from the tissues described above and were gated as shown. (b) ISM1^+^ cells from mouse lungs and isotype control are shown. (c) Statistical analysis of the percentage of ISM1^+^ cells in mouse lung compared with other tissues with lymphoid populations. Data shown are mean plus SEM, *n* = 7. Statistical analysis was performed by one-way ANOVA, ^∗∗∗^*p* < 0.0001.

To better characterize ISM1^+^ cells, we focused our study in the lung, which is one of the tissues with the highest mRNA expression of ISM1, both in mouse and human [18, 19]. To this end, we performed flow cytometry analyses on lung cells and confirmed that around 20% of them were ISM1^+^ (Figure 1(a)). Compared to other immunological tissues (including lamina propria, bone marrow, and blood), we observed a 4-fold higher level of ISM1^+^ cells in the lung (Figure 1(b)).

**Figure 2:**
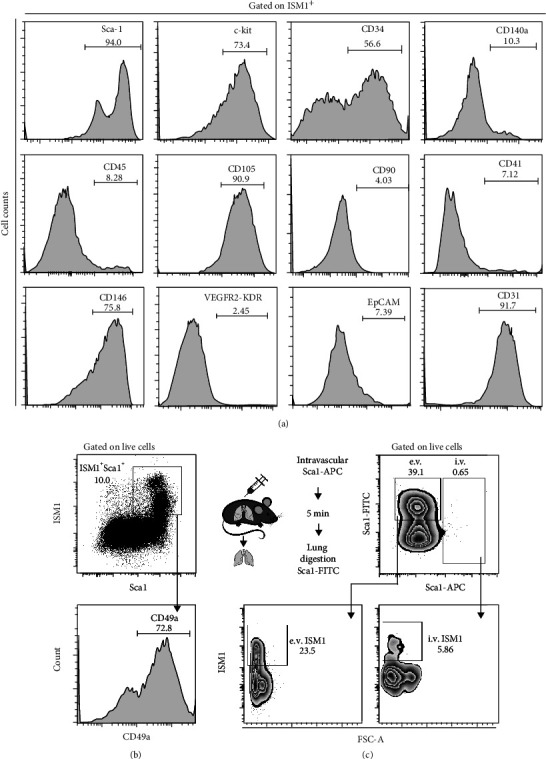
ISM1^+^ cells have markers of progenitor cells and are lung-resident cells. Lung-derived cells were stained with different antibodies, and histograms of each marker are depicted. A representative experiment is shown of at least 3 independent experiments. Whole ISM1^+^ cells were selected as in Figure 1. (b) Lung cells were stained with antibodies to label ISM1, Sca-1, and CD49a. Percentages are shown. (c) WT mice were injected with APC anti-Sca-1, and after 5 min, cells were recovered from the lung and stained with FITC anti-Sca-1 and PE anti-ISM1. Percentages in intravascular and extravascular regions are shown. A representative experiment (out of 3) is shown.

Since the lung is a mucosal tissue with significant cell diversity, we sought to identify which cells produce ISM in this organ. To this end, we evaluated different cell types including hematopoietic, mesenchymal, endothelial, and epithelial cells. A small fraction of ISM1 expressing cells also coexpress CD45, indicating that a portion of ISM1^+^ cells have a hematopoietic origin. Interestingly, around 90% of ISM1^+^ cells coexpressed high levels of Sca-1, CD105, CD146, and CD31, markers found in hemogenic endothelium and mesenchymal cells [20, 21] (Figure 2(a)). Furthermore, a fraction of ISM1^+^ cells also expressed c-kit and CD34, which represent markers of hematopoietic progenitors [22].

Our data also revealed that a small proportion of ISM1^+^ cells may be endothelial or epithelial cells, since we found low percentages of ISM1^+^ cells expressing VEGFR2 [23] and EpCAM (Figure 2(a)) [24].

### 3.2. ISM1^+^ Cells Have Properties of Lung Resident Cells

Stem cells have properties of resident cells. We therefore investigated the presence of CD49a, an integrin expressed by cells that bind extracellular matrix in the lung [25], and we observed that the majority of ISM1^+^ Sca-1^+^cells coexpressed CD49a (Figure 2(b)). We then took advantage of the fact that the majority of ISM1^+^ cells coexpressed Sca-1 (Figure 2(a)) and determined the relative proportions of intravascular and extravascular ISM1^+^ Sca-1^+^ cells by using an intravascular labeling technique previously described [13]. We observed that the majority of lung Sca-1^+^ cells were located in the extravascular compartment and coexpressed ISM1 (Figure 2(c)). Interestingly, we also identified a small subset of Sca-1^+^ ISM1^+^ in the intravascular compartment, indicating the presence of circulating ISM1^+^ cells as we previously observed (Figure 1(b)). Taken together, our data suggest that ISM1^+^ cells are resident cells of the lung.

### 3.3. ISM1 Identifies Subsets of Hematopoietic, Epithelial, and Mesenchymal Progenitor-Like Cells

**Figure 3:**
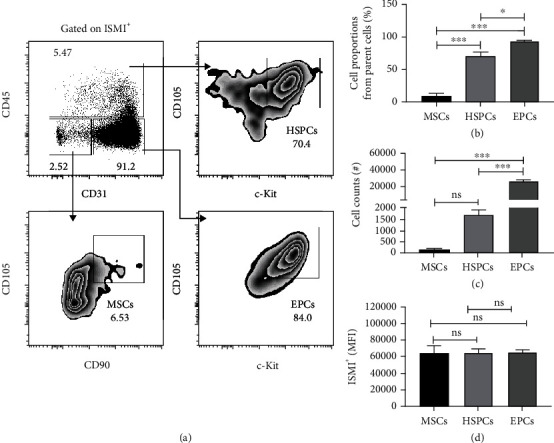
ISM1^+^ cells have a phenotype of progenitor cells related to endothelial, mesenchymal, and hematopoietic cells in the lung. (a) Lung whole ISM1^+^ cells were selected based on its CD45, CD31, CD105, c-kit, and CD90 expression, and subpopulations are shown. HSPCs (ISM1^+^CD45^+^CD31^−^CD105^+^c-kit^+^), MSCs (ISM1^+^CD45^−^CD31^−^CD105^+^CD90^+^), and EPCs (ISM1^+^CD45^−^CD31^+^CD105^+^c-kit^+^) are depicted. (b) Cell proportions of each subpopulation are plotted. (c) Total events corresponding to each subpopulation are plotted. (d) MFI of ISM1^+^ cells within each subpopulation are shown. Data shown are the mean plus SEM, *n* = 4. Statistical analysis was performed by one-way ANOVA, ^∗^*p* < 0.05, ^∗∗∗^*p* < 0.0001.

Our data suggest that ISM1 production is strongly associated with cells expressing progenitor-cells-associated markers. To further clarify this, we performed a multiparametric panel analysis to determine the presence of ISM1 in EPCs [26], MSCs [27], and HSPCs [13] subpopulations as described. As shown in Figure 3, ISM1^+^ cells were mainly EPCs (ISM1^+^CD45^−^CD31^+^CD105^+^c-kit^+^) and HSPCs (ISM1^+^CD45^+^CD31^−^CD105^+^c-kit^+^), although some ISM1^+^ cells resemble classic MSCs (ISM1^+^CD45^−^CD31^−^CD105^+^CD90^+^).

**Figure 4:**
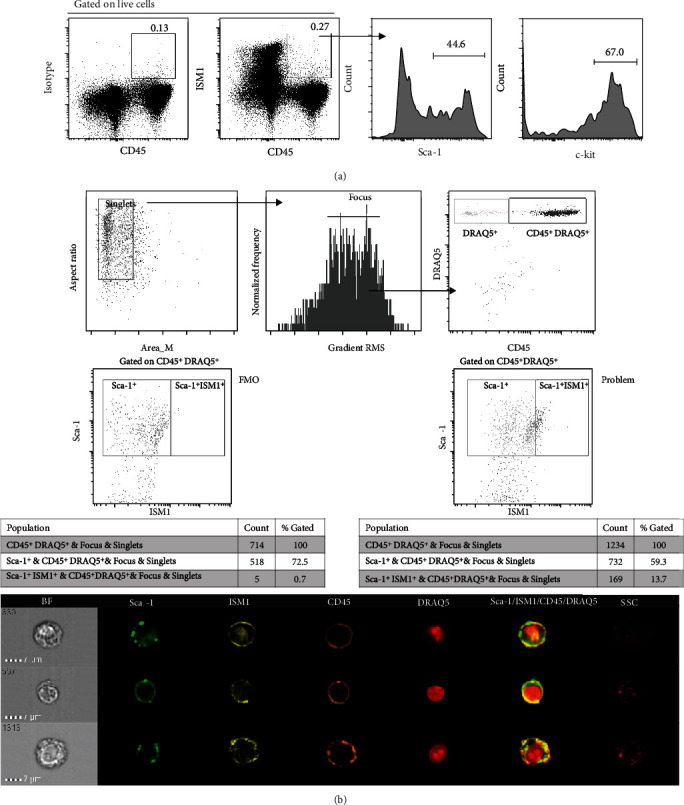
ISM1^+^ cells include hematopoietic progenitor stem cells. (a) Lung-derived ISM1^+^CD45^+^ cells were selected and analyzed for Sca-1 and c-kit coexpression. (b) ImageStream analysis of Sca-1^+^CD45^+^ISM1^+^ lung cells. For (a) and (b), a representative experiment (out of 3) is shown. FMO: fluorescence minus one control.

Since we found that ISM1^+^ cells share markers with already described lung HSPCs [13], we suspected that some lung hematopoietic stem cells may be characterized by the expression of ISM1. As shown in Figure 4, a fraction of ISM1^+^ cells coexpress CD45, Sca-1, and c-kit, further supporting the conclusion that ISM1 is expressed by a subpopulation of lung HSPCs. We confirmed the coexpression of CD45, Sca-1, and ISM1 by ImageStream in single cells (Figure 4(b)).

### 3.4. A Subset of ISM1^+^ Cells are Likely Hematopoietic Progenitors

**Figure 5:**
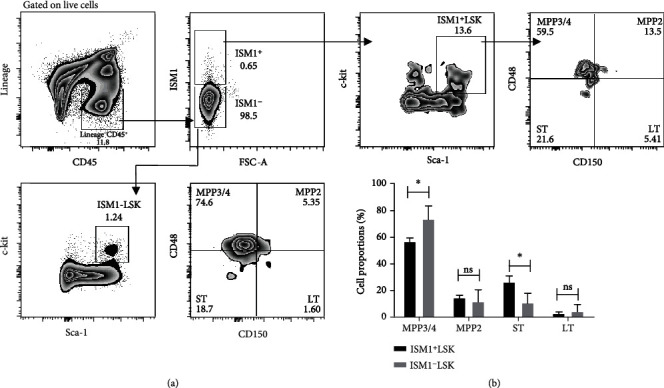
Characterization of ISM1^+^ LSK cells. (a) Gating strategy to visualize ISM1^+^ LSK (LIN^−^ CD45^+^ ISM1^+^ c-kit^+^ Sca-1^+^) and ISM1^−^ LSK (LIN^−^ CD45^+^ ISM1^−^ c-kit^+^ Sca-1^+^) cell subsets. LIN^−^CD45^+^ISM1^+^ cells were analyzed for the presence of Sca-1 and c-kit and then analyzed for CD150 and CD48 expression. (b) Percentages of different HSCs and progenitors are depicted, *n* = 3. Data are mean plus SEM *n* = 3 independent experiments. Statistical analysis was performed one-way ANOVA test, ns: nonsignificant; ^∗∗^*p* < 0.01, ^∗∗∗^*p* < 0.001.

The diversity of HSPCs characterized by the expression of Sca-1 and c-kit and by members of the SLAM family such as CD48 and CD150 has already been identified in the lung. Therefore, we sought to determine whether ISM1^+^ was also expressed by those subpopulations. We characterized hematopoietic progenitors and stem cells by following a flow cytometry gating strategy previously reported for lung HSCs [13]. We analyzed the Lineage^−^ CD45^+^ ISM1^+^ and Lineage^−^ CD45^+^ ISM1^−^ subsets, then we also looked for the presence of Sca-1 and c-kit expression to obtain a fraction of cells we called ISM1^+^LSK (Lineage^−^ CD45^+^ ISM1^+^ c-kit^+^ Sca-1^+^) or ISM1^−^ LSK (Lineage^−^ CD45^+^ ISM1^−^ c-kit^+^ Sca-1^+^). We observed that Lineage^−^ CD45^+^ ISM1^+^ cells represented around 1% of all Lineage^−^ CD45^+^ cells, and, interestingly, these ISM1^+^LSK cells express CD150 and CD48, indicating that phenotypically they are hematopoietic progenitors that can be classified as LT and ST-HSCs and MPP2-4 cells (Figure 5(a)). Furthermore, the ISM1^−^ LSK cells represented the classical LSK subset since they comprised around 99% of the Lineage^−^ CD45^+^ analyzed cells, and they also expressed CD150 and CD48 (Figure 5(a)). However, when we analyzed the different HSC cell subsets found in ISM1^+^LSK and ISM1^−^LSK populations, we detected a higher proportion of ST-HSC precursors in the ISM1^+^LSK compartment. Conversely, we observed a higher proportion of MPP3/4 precursors in the ISM1^−^LSK compartment. We found no significant differences in the proportions of LT and MPP2 (Figure 5(b)). Considering that ST-HSCs are above MPP3/4 precursors (in the hierarchy of stemness properties) [22], our data suggest that ISM1^+^LSK cells represent a subset of hematopoietic progenitors enriched in a compartment of less-differentiated cells than those located in the ISM1^−^LSK compartment.

### 3.5. Changes in the Compartment of Lung LSK and ISM1^+^LSK Cells during Bacterial Infection

**Figure 6:**
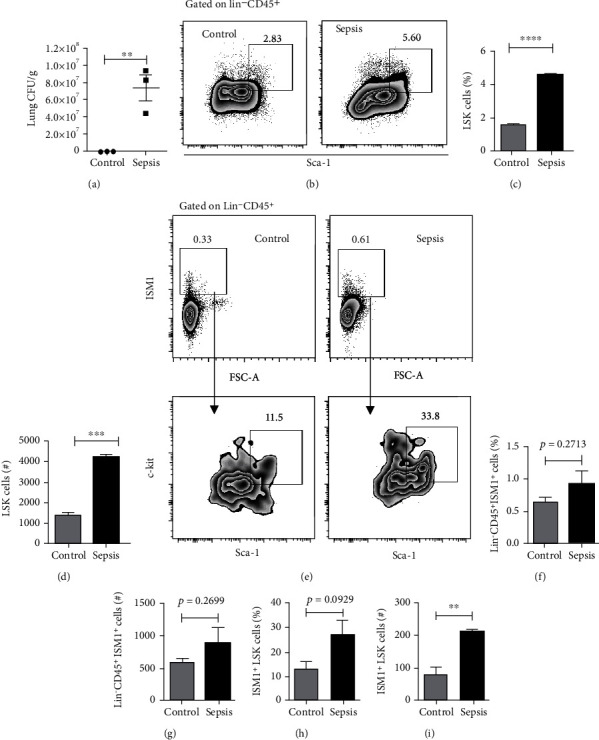
Changes in LSK and ISM1^+^LSK cell subsets after *P. aeruginosa* challenge. (a) CFU from lung tissues after *P. aeruginosa* infection. (b) LKS cells (Lineage-CD45^+^c-kit^+^Sca-1^+^) from control and septic mice were analyzed, and percentages (c) and absolute numbers (d) are depicted. (e) ISM1^+^LSK cells were analyzed, and percentages and absolute numbers are depicted (f–i). Data are means plus SEM *n* = 3 independent experiments. Statistical analysis was performed by Student's *t*-test for unpaired data, ^∗∗^*p* < 0.01, ^∗∗∗^*p* < 0.001, ^∗∗∗∗^*p* < 0.0001.

An important property of bone marrow HSCs is their capacity to proliferate during emergencies caused by pathogens [28]. However, it is still unknown whether lung HSCs respond in the same way as their bone marrow counterparts during bacterial challenges, and more importantly, whether the ISM1^+^-HSC compartment identified in this study is similarly affected. To answer this, we used a mouse model of sepsis generated by IP inoculation of the opportunistic bacterial pathogen *P. aeruginosa*, and 8 h after infection, we successfully detected bacteria in the lungs (Figure 6(a)). While we did not detect significant changes in the analysis of total lung cells (data no shown), we observed small increases in the percentage, absolute number, and mean intensity fluorescence of CD45^+^ISM1^+^ lung cells from infected mice (Figure S1). We hypothesized that this outcome could be associated with an increase in the hematopoietic progenitor cell subset. To explore this, we analyzed the LSK compartment and confirmed that bacterial infection increased the percentage and absolute numbers of LSK cells (Figures 6(b)–6(d)). Therefore, bacterial infection changes the lung HSC compartment in a similar fashion as its bone marrow counterpart [28]. Next, we sought to evaluate whether the hematopoietic ISM1^+^ cell compartment was also affected. We initially observed a slight increase in the percentage and absolute numbers of Lin^−^ CD45^+^ ISM1^+^ cells (Figures 6(e)–6(g)). Furthermore, when we analyzed the ISM1^+^LSK subset (Lin^−^ CD45^+^ ISM1^+^ c-kit^+^ Sca-1^+^), we found that the absolute numbers of ISM1^+^LSK cells were significantly increased (Figure 6(i)), indicating that ISM1^+^-HSC cells are also altered during bacterial infection.

**Figure 7:**
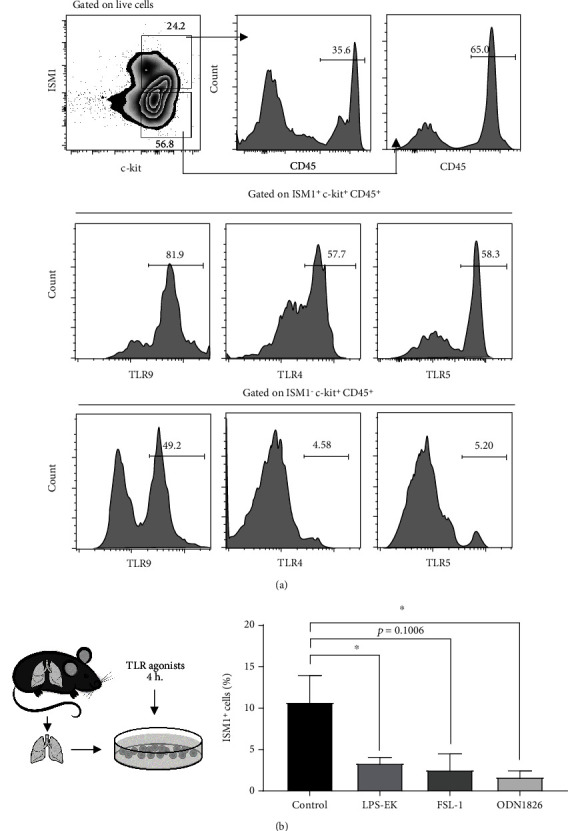
Lung ISM1^+^ progenitor-like cells express TLR9, TLR4, and TLR5. (a) Whole lung cells were stained against ISM1, c-kit, and CD45, and TLRs presence were detected. A representative experiment is shown out 3 independent experiments. (b) *In vitro* stimulation of lung cells with TLR9, TLR4, and TLR5 agonists; ISM1^+^ cells decreased after 4 h of stimulation. Data are means plus SEM *n* = 3 independent experiments. Statistical analysis was performed by one-way ANOVA; ^∗^*p* < 0.05.

The lung is a target of *P. aeruginosa*, and its products have been reported to stimulate HSPCs through Toll-like receptors (TLRs), particularly TLR4 [28]. However, TLR5 and TLR9 are also involved in lung infections [29–31]. We therefore examined the presence of these TLRs on ISM1^+^c-kit^+^CD45^+^ lung cells. We observed that around 50% of ISM1^+^c-kit^+^CD45^+^ express TLR4 and TLR5 and approximately 90% express TLR9 compared to ISM1^−^c-kit^+^CD45^+^ cells (Figure 7(a)). Furthermore, *in vitro* stimulation of purified lung cells with TLR4, -5, and -9 agonists reduced the percentage of ISM1^+^ cells (Figure 7(b)), suggesting that ISM1^+^ cells have the molecular pathways required to respond to bacterial challenges.

## 4. Discussion

*ISM1* is a highly conserved gene among species [16], and it is highly expressed in barrier tissues including the respiratory track, mucosal tissues, and hematopoietic cells [19]. We previously reported that immune cells located in the lung, including T and NK cells, expressed ISM1[19]. However, a more comprehensive characterization of the cells expressing this secreted protein has not been done.

By evaluating the presence of intracellular soluble ISM1 in the lungs and other immune tissues including lamina propria, bone marrow, and blood, we confirmed that ISM1^+^ cells are enriched in the lung as mRNA expression has suggested [18, 19]. Furthermore, the enrichment of ISM1^+^ cells in the lung suggests that the lung milieu may be important for the maintenance of ISM1^+^ cells.

ISM1 is expressed by mesodermal tissues during development [16–18]. However, previous studies have described a strong presence of ISM1 lining lung's bronchi, suggesting that ISM1 may be associated with epithelial cells [18, 32]. Interestingly, we found a small percentage of ISM1^+^ cells expressing EpCAM, a *bona fide* marker of epithelial cells [24]. This observation suggests that ISM1 expression may be reduced in differentiated epithelial cells. Thus, we hypothesized that ISM1 may be expressed by a subtype of mesodermal-derived cell population. Since endothelial cells [33], mesenchymal cells [34], and hematopoietic progenitor cells [35] are derived from mesoderm, we confirmed the presence of ISM1 in cells coexpressing Sca-1, CD105, CD146, c-kit, CD34, and CD45, suggesting that ISM1 expression may be a novel biomarker of progenitor-like cells. Furthermore, the presence of CD49a on Sca-1^+^ISM1^+^ cells (Figure 2) (along with their extravascular localization) suggests that these cells are tissue residents and require signals from a niche located in the lung (which remains uncharacterized) [36].

Our data strongly suggest that ISM1^+^ cells may help to identify EPCs (ISM1^+^CD45^−^CD31^+^CD105^+^c-kit^+^) and HSPCs (ISM1^+^CD45^+^CD31^−^CD105^+^c-kit^+^) and in minor degree MSCs (ISM1^+^CD45^−^CD31^−^CD105^+^CD90^+^) (Figure 3). Thus, ISM1 may identify endothelial-like and mesenchymal-like cells that are part of a lung stromal niche [37]. Furthermore, since the majority of ISM1^+^ lung cells seem to be EPCs akin to zebra fish stroma [38], ISM1 could have a similar key role in the support of hematopoiesis. This is a possibility that deserves further studies [17].

Mucosal tissues are important reservoirs of hematopoietic progenitors [3, 39, 40]; thus, we performed a detailed analysis of ISM1^+^ cells in the context of HSPCs. We found that ISM1 is present in a fraction of LSK cells (ISM1^+^ LSK cells) that coexpress CD150 and CD48, revealing a novel subpopulation of ISM1^+^ cells enriched in the compartment of ST-HSCs progenitors which have elevated differentiation potential [22]. Since ISM1 is required for normal hematopoiesis in zebrafish [17], this novel subpopulation of ISM1^+^-HSPC cells may be important for the regulation of extramedullary hematopoiesis in the lung. Although the function of ISM1 in the HSPC compartment remains a matter of further studies, ISM1 may help to maintain the lung HSPC niche given its relationship with the TGF-*β* superfamily through the NODAL/Activin axis [41, 42].

Acute inflammation due to bacterial challenges or injuries has been demonstrated to activate HSPCs in the bone marrow [43]. Inflammatory mediators including IL-6 [44], TNF-*α* [45], IFN-*α* [46], IFN-*γ* [47], TGF-*β* [48], and M-CSF [49] regulate HSC activation, mobilization, and proliferation. Such mediators are secreted upon a pathogen response and promoted the mobilization of HSPCs within the bone marrow [50]. However, it is unknown whether the lung HSPCs described by Lefrançais et al. [13] share the same response properties as bone marrow HSPCs. Our data indicates that systemic challenge with *P. aeruginosa* amplified total lung LSK cell counts and proportions (Figure 6). This finding strongly suggests that lung HSPCs act similarly to bone marrow LSK cells under bacterial infections like *P. aeruginosa* [28], *M. tuberculosis* [51], and *E. muris* [52]. Importantly, the absolute numbers of ISM1^+^ LSK cells were increased too, suggesting that, *in vivo*, bacterial challenges elicit an expansion of ISM1^+^ LSK cells.

It is well known that both mature and immature hematopoietic cells can be directly stimulated by pathogen-associated molecular pattern (PAMP) recognition due to the activation of pathogen recognition receptors (PRRs) such as Toll-like receptors [50, 53]. We wanted to know whether lung ISM1^+^ cells expressed some common TLRs that could account for the effects observed by the bacterial challenge. As shown in Figure 7, we found that ISM1^+^c-kit^+^CD45^+^ expresses the most common TLR in the lungs, TLR9, TLR4, and TLR5 [54], in analogy to bone marrow HSPCs [55]. Direct stimulation of those TLRs *in vitro* resulted in a decrease of ISM1^+^ cells, suggesting that TLR stimulation may trigger the secretion of ISM1 from lung cells and therefore explaining the increased levels of ISM1 in bronchoalveolar lavage that were observed in a mouse model challenged with intratracheal LPS [56].

These observations are potentially important given the emerging importance of the lung as a site for hematopoiesis. For example, Lefrancais et al. (2017) have reported that the lung is a particularly important site of platelet biogenesis and may account for approximately 50% of total platelet production or 10 million platelets per hour. In this context, future studies may focus on a potential role for ISM1 in platelet biogenesis, among other important aspects of potential hematopoiesis in the lung.

## 5. Conclusions

Taken together, our study demonstrates that ISM1 is expressed by several lung cell subsets. Some of these have a phenotype of progenitor cells and also exhibit properties of lung tissue resident cells. Thus, ISM1 is likely a novel biomarker associated with progenitor-like cells. Moreover, we identified a novel subset of ISM1^+^-ST-HSCs in the compartment of LSK cells. Finally, bacterial infection increased both LSK and ISM1^+^LSK cell numbers, most likely through activation of TLRs.

Overall, our data strongly suggests that ISM1 has a role in hematopoiesis in the lung, specifically, through the physiology of lung HSPCs. Consequently, a detailed exploration of the function of ISM1 in lung extramedullary hematopoiesis during both homeostasis and infection/inflammation will help to define the function(s) of ISM1 in this emerging area.

## Data Availability

Data is available upon request (contact: Valle-Rios R email: vallerios@unam.mx).

## Supplementary Materials

Supplementary 1 Figure S1: changes in ISM1^+^CD45^+^ cells after *P. aeruginosa* challenge. (A) Lung cells were stained with antibodies that recognize ISM1 and CD45 following *P. aeruginosa* challenge. Percentages (B), absolute numbers (C), and MFI (D) are shown. A representative experiment (out of 3) is shown. Table S1: list of antibodies. General characteristics of the antibodies used during experimental procedures. Working dilutions are depicted.
