# Supplementary material for: Isthmin 1 is Expressed by Progenitor-Like Cells in the Lung: Phenotypical Analysis of Isthmin 1+ Hematopoietic Stem-Like Cells in Homeostasis and during Infection
Source: J Immunol Res. 2022 Apr 1;2022:2909487. doi: 10.1155/2022/2909487 (PMC8993550; doi:10.1155/2022/2909487)
Supplement: Supplementary 1 — Figure S1: changes in ISM1+CD45+ cells after P. aeruginosa challenge. (A) Lung cells were stained with antibodies that recognize ISM1 and CD45 following P. aeruginosa challenge. Percentages (B), absolute numbers (C), and MFI (D) are shown. A representative experiment (out of 3) is shown. Table S1: list of antibodies. General characteristics of the antibodies used during experimental procedures. Working dilutions are depicted. [file 2909487.f1.pdf]

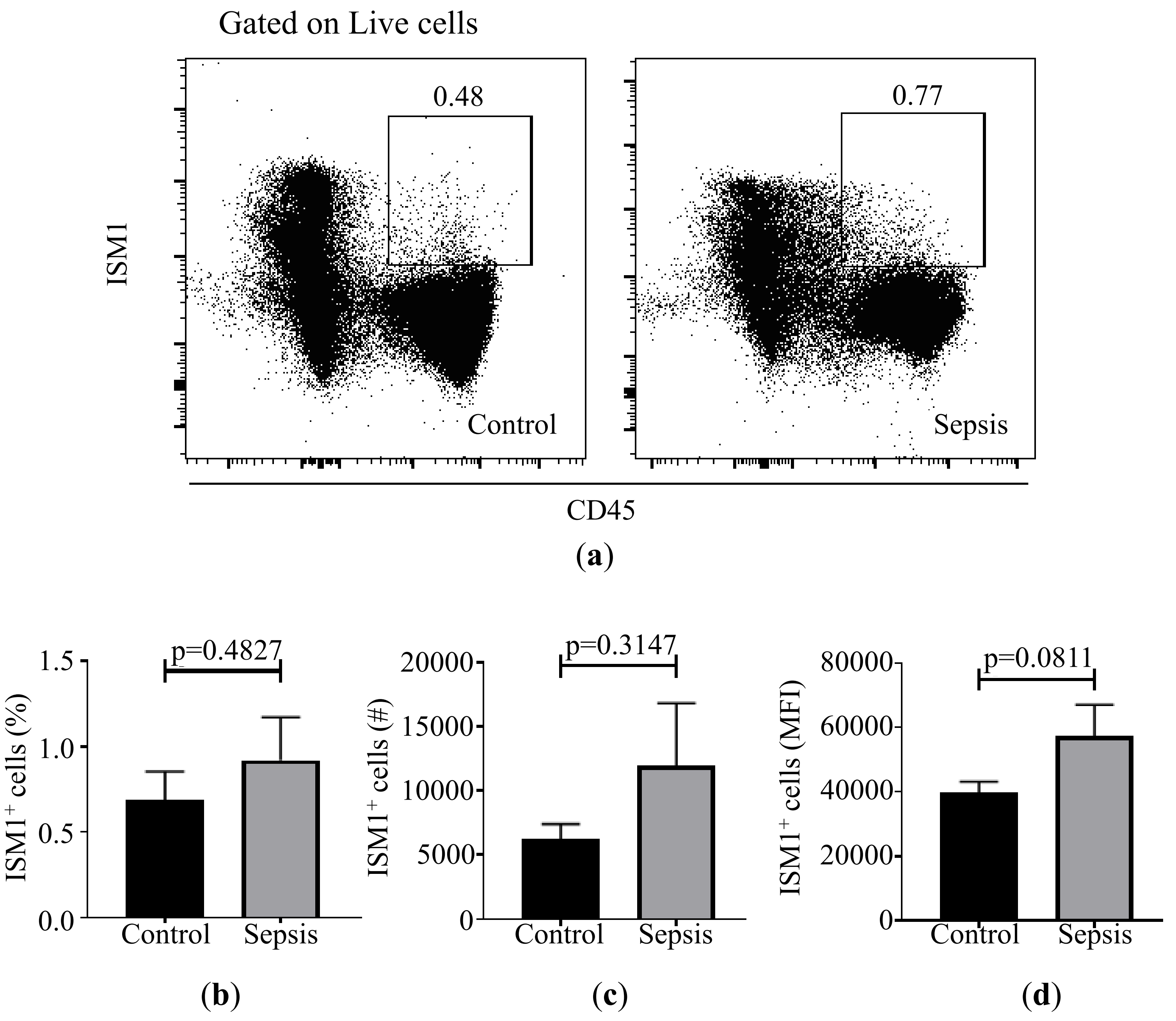

Fig. S1. Changes in ISM1^{+}CD45^{+} cells after *P. aeruginosa* challenge. (a). Lung cells were stained with antibodies that recognize ISM1 and CD45 following *P. aeruginosa* challenge. Percentages (b), absolute numbers (c), and MFI (d) are shown. A representative experiment (out of 3) is shown.

Marker	Antigen	Fluorochrome	Reactivity	Clone	Brand	Dilution
Homing integrins	CD49A	APC	Mouse	HMA1	Biolegend	3:200
	CD49B	EF-450	Mouse	DX5	Ebioscience	2:200
	CD49D	FITC	Mouse	R1-2	Biolegend	2:200
Leukocyte common antigen	CD45	AF700	Mouse	30F11	Biolegend	1:200
	CD45	APC	Mouse	30F11	Biolegend	1:200
T cell	CD3E	PERCP-CY5.5	Mouse	145-EC11	Tonbo	5:200
B cell	CD19	PERCP-CY5.5	Mouse	1D3/CD19	Biolegend	5:200
T cell $CD8^+$	CD8A	PERCP-CY5.5	Mouse	53-6.7	Biolegend	5:200
T cell $CD4^+$	CD4	PE	Mouse	GK1.5	Ebioscience	4:200
	CD4	PE-CY7	Mouse	GK1.5	Ebioscience	4:200
	CD4	AF488	Mouse	GK1.5	Ebioscience	4:200
	CD4	APC-CY7	Mouse	GK1.5	Ebioscience	4:200
	CD4	PERCP-CY5.5	Mouse	GK1.5	Ebioscience	4:200
Erythrocytes	TER119	PERCP-CY5.5	Mouse	TER119	Biolegend	5:200
Mast cells	FcRε1A	PERCP-CY5.5	Mouse	MAR-1	Biolegend	5:200
Monocytes/Macrophages	CD11B	PERCP-CY5.5	Mouse/Human	M1-70	Tonbo	5:200
Dendritic cells	CD11C	PERCP-CY5	Mouse	N418	Biolegend	5:200
Natural killers	NK1.1	PERCP-CY5.5	Mouse	PK136	Biolegend	3:200
Innate lymphoid cells	ST2	APC	Mouse	DIH9	Biolegend	3:200
	CD335 (NKp46)	BV421	Mouse	29A1.4	Biolegend	3:200
	CD127 (IL7R)	VF450	Mouse	A7R34	Tonbo	3:200
Mesenchymal and stromal cells	CD90.2	AF700	Mouse	30-H12	Biolegend	0.05
	CD105	FITC	Mouse	MJ7/18	Biolegend	3:200
	CD146 (MCAM)	APC	Mouse	ME-9F1	Biolegend	3:200
	CD140a (PDGFRa)	BV605	Mouse	APA5	Biolegend	3:200
Epithelial	CD326 (EpCAM)	PERCP-CY5.5	Mouse	G8.8	Biolegend	3:200
Endothelial	CD31 (PECAM-1)	PE-CY7	Mouse	MEC13.3	Biolegend	3:200
HPSCs	CD117 (ckit)	APC750	Mouse	2B8	Biolegend	3:200
	SCA-1	FITC	Mouse	E13-161.7	Biolegend	3:200
		APC	Mouse	E-13-161.7	Biolegend	3:200
	CD34	PERCP-CY5.5	Mouse	HM34	Biolegend	5:200
	CD48	APC-fire	Mouse	HM48-1	Biolegend	2.5:200
	CD150	VB605	Mouse	A12 (7D4)	Biolegend	3:200
Isthmin-1	ISM1	PE	Mouse	B054B6	Biolegend	1.5:50
Isotype control for anti-Isthmin-1	IgG2b	PE	Mouse	MPC-11	Biolegend	1.5:50
GRP78/Bip	GRP78	AF488	Mouse/Human	C38	Ebioscience	3:200
TLRs	TLR4	PECy7	Mouse	SA15-21	Biolegend	3:200
	TLR5	AF647	Mouse	ACT5	Biolegend	3:200
	TLR9	FITC	Mouse	S18025A	Biolegend	2:200

Table S1. List of antibodies. General characteristics of the antibodies used during experimental procedures. Working dilutions are depicted.
